# Effect of Static Magnetic Field on the Quality of Pork during Super-Chilling Storage

**DOI:** 10.3390/foods13081205

**Published:** 2024-04-16

**Authors:** Ting Wang, Yamei Jin, Xiao Zhang, Na Yang, Xueming Xu

**Affiliations:** 1 State Key Laboratory of Food Science and Technology, Jiangnan University, Wuxi 214122, China; w1961599381@163.com (T.W.); yameiking123@126.com (Y.J.); 19855651302@163.com (X.Z.); xmxu@jiangnan.edu.cn (X.X.); 2 School of Food Science and Technology, Jiangnan University, Wuxi 214122, China; 3 Collaborative Innovation Center of Food Safety and Quality Control in Jiangsu Province, Jiangnan University, Wuxi 214122, China

**Keywords:** pork, static magnetic field, super-chilling, physicochemical properties, myofibrillar proteins, storage

## Abstract

Fresh pork tenderloin was stored at −3 °C under different static magnetic fields (SMF) of 0, 4, and 10 mT (control, MF-4, and MF-10) to investigate their physicochemical properties changes during storage of 8 days. The initial equilibrium temperature of the samples stored with 4 mT MF was found to be −2.3 °C, which was slightly lower (0.3 °C) than that the control value. The super-chilling phenomenon on the pork was then observed, as the samples stored under the magnetic field did not freeze throughout storage period, but the control experienced a sudden change in temperature after 138 h and then froze. The preservation effect of MF-4 on meat quality was the best in all treatment groups. MF-4 achieved a higher water-retention rate, with drip and cook losses of 6.5% and 29.0% lower than the control, respectively. Meanwhile, the MF-4 effectively delayed the color change in the meat during the storage and the texture hardening after cooking, and effectively controlled the growth of the total volatile saline nitrogen content on the samples. In addition, MF-4 delayed the reduction in myofibrillar protein solubility, sulfhydryl content, and emulsification capacity, indicating that this field inhibited the denaturation of myofibrillar protein. This study can be considered as an application reference of magnetic fields during meat storage at a super-chilled temperature.

## 1. Introduction

Super-chilling is also known as partial freezing, in which the product temperature is lowered to about 1–2 °C below the initial freezing temperature [1]. The super-chilling process is completed in two stages; in the first stage, the product temperature is lowered to the freezing point (pre-cooling), while in the second stage, the latent heat of crystallization is removed (phase-transition) [2]. At super-chilled state, the food surface freezes, forming a thin layer of ice, while the internal and external temperatures of the food eventually equilibrate during subsequent storage [1,3], resulting in a reduction in energy costs [4]. As for super-chilled foods, only a partial freezing (5–30%) of the sample happens [2] and the shelf life of the food is extended by 1.5 to 4.0 times compared to the refrigerated foods. On the other hand, although the shelf life of super-chilled foods is shorter than that of frozen food, super-chilling is gentle to the foods, showing lower protein denaturation and less structural damage [5].

The growth of microorganisms and their proliferation are important factors to affect food quality and shelf life. Many studies have shown that the super-chilling can effectively inhibit microbial growth and biochemical reactions in meat [6,7], in turn extending their shelf life. Super-chilling also reduces the degree of myoglobin oxidation, retaining the meat color [8,9]; exhibits lower fat oxidation [6,10,11]; and inhibits protein hydrolysis [12,13]. However, there are certain negative aspects of super-chilled storage, including larger ice crystal formation. Bahuaud et al. [14] found the larger ice crystals formed outside and inside the muscle fibers during super-chilling disrupted the structural integrity of the fillets, resulting in an increased separation between muscle fibers with an increased myofibril fracture. In addition, the partially partly frozen water leads to an increment in solute concentration, both inside and outside the cells, which facilitates certain biochemical reactions during the storage, leading to pH changes and protein denaturation [15]. The magnetic field being a physical factor, its weak magnitude has the characteristics of excellent biocompatibility and magnetic penetrability into the food and has been continuously applying in food freezing [16,17]. Studies have shown the influence of the magnetic field on the morphology of ice crystals formed inside the frozen meat, which leads to less damage to the cell membrane, resulting in a better quality of the thawed products to some extent. A permanent magnetic field (20 mT) and an alternating magnetic field (1.26 mT) were found to reduce the area of ice crystal by 67% and 79%, respectively, during freezing of cherries while forming uniform ice crystals [18]. Gan et al. [19] compared the effect of static MFs with different strength ranging from 0 to 10 mT during pork freezing and the highest freezing rate was achieved at the strength of 6 mT, while an improvement in meat quality resulted through forming smaller ice crystals, reducing thawing and cook losses, and avoiding damage to myofibrillar proteins. Applying a magnetic field during the freezing of cucumber tissue liquid and steak can also reduce nucleation temperature and inhibit the formation of ice crystals [20,21].

The magnetic field has been used during cold storage and freezing; however, to the best of our knowledge, there are relatively few studies on its use in the super-chilling preservation of pork to see its effect on quality characteristics. Therefore, we studied the influence of magnetic field during pork freezing to see its super-chilling effect. The freezing point of pork tenderloin is −1.5 °C [16]. We choose −3 °C as the storage temperature for the super-chilled storage of pork. Two static magnetic fields (4 and 10 mT) were investigated for the preservation of the pork, while the freezing sample without magnetic field was considered as the control. The super-chilled pork quality was judged using physical characteristics (freezing behavior, water holding capacity, texture, and color), chemical properties (TVBN and myofibrillar protein) at 2, 4, 6, and 8 days of the storage. Thus, the present work aimed to study the effect of static MFs on inhibiting pork freezing and improving the storage quality of pork.

## 2. Materials and Methods

### 2.1. Sample Preparation

Pork tenderloins, slaughtered that day, were purchased from a slaughter house (Wuxi, Jiangsu, China). All the muscles were immediately put into portable chilled boxes (4–6 °C) and transported to the laboratory within 2 h after killing. All the visible fat and connective tissue was removed before the tenderloin was cut along a direction parallel to the muscle fiber axis into cubes measuring 3.5 × 3.5 × 3.5 cm^3^, which weighed approximately 50 g. They were then kept at 4 °C for 8 h (pre-cooling). The super-chilling preservation was performed at −3 °C by using a freezer, connected with magnetic field (INDUC Scientific Co., Ltd., Wuxi, China; MFF10). In the experiments, the samples were kept at the constant-temperature chamber with a uniform magnetic field of 4 mT (MF-4) and 10 mT (MF-10), while the sample kept without magnetic field was used as the control. Samples were collected for analysis at 2 d, 4 d, 6 d, and 8 d of storage, and the super-chilled samples were transferred to chilling condition (4 °C) for 3 h before analysis.

### 2.2. Magnetic Field Device

**Figure 1:**
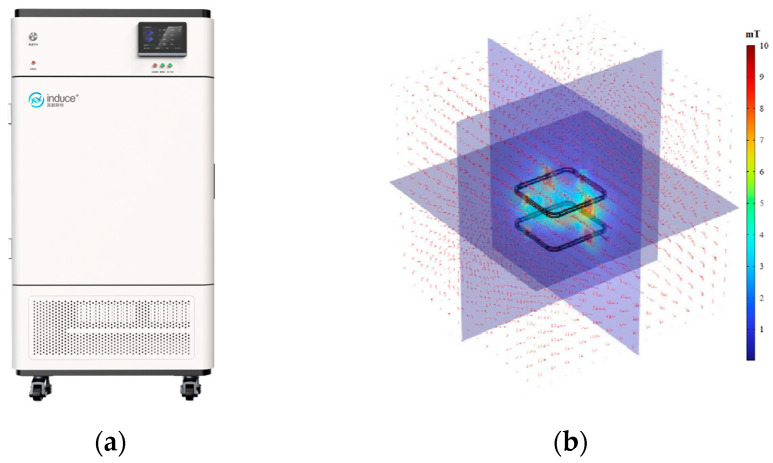
Magnetic field freezer equipment diagram (**a**) and numerical simulation of magnetic field in sample chamber (**b**).

The generation of magnetic field was conducted by the method described by Wang et al. [22] with minor modifications to the parameters. A magnetic field freezer (MFF10, INDUC Scientific Co., Ltd., Wuxi, China) was used for super-chilling storage, which contains a magnetic-field generator, sample chamber, refrigeration unit, fan, and control panel (Figure 1a). The magnetic-field generator consisted of a pair of Helmholtz coils (80 cm × 80 cm square; 400 turns) and a power supply, generating a uniform magnetic field of 0–10 mT at an excitation current of 0–8 A. The sample chamber dimensions were 38 cm × 32 cm × 40 cm, and the cooling temperature range was from −20 °C to room temperature (25 °C). The intensity and uniformity of the magnetic field (99%) in the sample were tested by Gaussian meter (BLD-1030, Bolandun Co., Ltd., Beijing, China). In addition, the COMSOL multiphysics 6.2 software package was used to numerically simulate the magnetic field in sample chamber (Figure 1b).

### 2.3. Freezing Curve

A T-type thermocouple was used along with a data logger (KSB10A0R, Ningbo Keshun Instrument Co., Ltd., Ningbo, Zhejiang, China) to record the temperature profile inside the sample at every 5 min by inserting the probe at the sample geometric center, which were used to plot the freezing curve. Three pieces of pork were tested under each storage condition, and use the average temperature value to plot the freezing curve.

### 2.4. Drip Loss

Following the method of Park et al. [23] with slight modification, drip loss was analyzed. Excess drips were removed from the surface of the sample before analysis. Drip loss value was given by the weight loss during the measurement period, which was calculated as follows:

(1) $$Drip\;loss\;(\%)=(M_{0}-M_{1})/M_{0}\times 100$$

where *M*_0_ is the initial weight (g), and *M*_1_ is the final weight (g) of the pork.

### 2.5. Water Holding Capacity (WHC)

The weighted sample (about 2 g) was taken in a 50 mL centrifuge tube after wrapping in double-layer filter paper, while an adequate quantity of skimmed cotton was deposited at the bottom. After centrifugation at 4000 rpm for 10 min (4 °C), the sample weight was taken immediately after removing the filter paper. Equation (2) was used to calculate WHC [16].

(2) $$WHC\;(\%)=[1-\left(M_{0}-M_{1}\right)]/(M_{0}\times WC)\times 100$$

where *M*_0_ is the initial mass (g), and *M*_1_ is the final mass (g) of the sample, while *WC* is the proportion of the moisture in each sample before centrifugation.

### 2.6. Cook Loss

Following the method of Jia et al. [24], cook loss was determined with minor modification. In brief, the sample was sealed in a plastic bag without the air and then heated at 85 °C for 20 min using a water bath. After cooling the sample to normal temperature (20 °C), the excess water was removed. Equation (3) was used to calculate cook loss formula.

(3) $$Cooking\;loss\;(\%)=\frac{m_{1}-m_{2}}{m_{1}}\times 100$$

where *m*_1_ and *m*_2_ are the sample weight before and after cooking, respectively.

### 2.7. Magnetic Resonance Imaging (MRI)

A low-field nuclear magnetic resonance (MesoMR23-060V-I, Suzhou, Jiangsu, China) was used for the MRI analysis [25] to allocate the moisture distribution and status of the sample. Then, the sample cubes (1 cm^3^) were inserted into an MRI sample tube. The slice width of 4.0 mm and slice gap of 1.0 mm were considered as the main parameters. The data were processed into pseudo-color images using the Neumay MRI image software Ver. 1.0 (Niumag Co., Ltd., Suzhou, Jiangsu, China) package to analyze the proton signal intensity.

### 2.8. Color Measurement

Samples were pre-cooled to 4 °C in a refrigerator before color measurement, and the surface of the sample was measured immediately after the excess water was removed from the surface. Instrumental color measurements included CIE L* (lightness), a* (redness/greenness), and b* (yellowness/blueness). Readings were performed using diffuse illumination, with D65 as illuminant and 10° as standard observer. Port size was 4 mm. The color values were determined using a chroma meter (Threenh Technology Co., Ltd., Shenzhen, Guangdong, China; NR110); the meter was calibrated with a standard plate before measurement [26] with minor modifications. During the measurement, determinations were made at six random places of the sample. Color difference value (∆*E*) was given by Equation (4).

(4) $$△E=\sqrt{{\left(L^{*}-{L_{0}}^{*}\right)}^{2}+{\left(a^{*}-{a_{0}}^{*}\right)}^{2}{\left(b^{*}-{b_{0}}^{*}\right)}^{2}}$$

where *L*_0_*, *b*_0_*, and *a*_0_* are the values for fresh samples; and L*, b*, and a* are the values for stored samples.

### 2.9. Texture Profile Analysis (TPA)

Following the method of Kim et al. [6], the sample was heated at 85 °C for 20 min using a water bath before texture analysis (Stable Micro Systems Co., Ltd., London, UK; TPA Texture Meter). A flat-bottom cylindrical probe (SMP P/25; 25 mm diameter) was used for the measurement through a double-compression cycle with 30% compression and 5 s interval; the trigger force used was 5 g. A pre-test speed of 2 mm/s, test speed of 1 mm/s, and post-test speed 2 mm/s were selected. The data were analyzed using the Exponent software package.

### 2.10. pH

The measurement of pH value is referred to Gan et al. [19], and slightly modified. The samples of 3 g pork sample was taken and added with 27 mL 0.1 mol/L KCl solution. The samples were beaten with a homogenizing beating machine for 60 s and then left for 5 min. The pH of the supernatant was determined with a pH meter (METTLER TOLEDO Instrument Co., Ltd., Shanghai, China).

### 2.11. Total Volatile Basic Nitrogen (TVBN)

The TVBN value was analyzed using the method of Zhang et al. [27] with minor changes. A grounded meat sample (10 g) was put into a conical flask containing distilled water (75 mL) and the mixture was shaken for half an hour, which was then filtered using a filter paper (two layers). The clear liquid so obtained was used for TVBN determination via an automatic Kjeldahl nitrogen analyzer (K1100F, Hanon Future Technology Group Co., Ltd., Jinan, Shandong, China).

### 2.12. Extraction of Myofibrillar Protein (MP)

Following the method of Park et al. [28] with minor changes, the extraction of MP was accomplished. The visible fat, fascia, and other tissues of the pork tenderloin were removed and minced. About 5 g of sample and 20 mL of buffer solution (containing 0.1 mol/L NaCl; 1 mmol/L EDTA; 2 mmol/L MgCl_2_; and 10 mmol/L phosphate buffer; pH 7) were homogenized for 1 min at 10,000 r/min. The homogenate was then centrifuged for 15 min at 4 °C using 2000× *g* force and the clear liquid was discarded. The solid precipitate was homogenized with 4 volumes (*w*/*v*) of sodium chloride wash solution (0.1 mol/L) and then centrifuged using the same homogenization and centrifugation parameters as above; here again, the clear liquid was discarded. The clear liquid was filtered with 4 layers of filter paper; the pH of which was adjusted to 6.0 with HCl (0.1 mol/L). The supernatant was discarded after centrifugation, and the resulting white paste was the myofibrillar protein; such extracted sample was kept at 4 °C for subsequent use. Then, 10 mL of phosphate buffer (containing 25 mmol/L phosphate buffer; and 0.6 mol/L NaCl; pH 7.0) was mixed with the precipitates. A BCA kit (Beyotime Biotechnology Co., Ltd., Shanghai, China) was used to determine the myofibrillar protein content.

### 2.13. Protein Solubility

Protein solubility was determined following the method of Du et al. [29] with minor modification. A 5 mL protein solution (5 mg/mL) prepared with 25 mmol/L phosphate buffer (pH 7.0) was taken in a 10 mL centrifuge tube, which was left for 2 h at 4 °C. After centrifugation (10,000 r/min) at 4 °C for 20 min, 1 mL of the clear liquid was taken to determine the protein concentration following the BCA (Bicinchoninic Acid Assay) kit instructions (Beyotime Biotechnology Co., Ltd., Shanghai, China). The solubility of protein was calculated using Equation (5).

(5) $$Protein\;solubility\;(\%)=(C_{1}{/C}_{0})\times 100$$

where *C*_0_ is the pre-centrifugation myofibrillar protein content (mg/mL); and *C*_1_ is the post-centrifugation myofibrillar protein content (mg/mL).

### 2.14. Reactive and Total Sulfhydryl Content

As described by Hou et al. [30], the sulfhydryl content (reactive and total) was analyzed with minor modification. An amount of 1 mL of protein solution (2 mg/mL) and 9 mL of buffer solution (50 mM phosphate buffer, containing 8 M urea, 0.6 M KCl, 10 mM EDTA (Ethylene Diamine Tetraacetic Acid), and 10 mM DTNB (5,5′-Dithiobis-(2-nitrobenzoic acid)); pH 7.0) were mixed at 10,000 r/min for 1 min. For the total sulfhydryl content, the mixture was incubated in an incubator (37 °C) for 30 min and centrifuged (10,000× *g* and 4 °C) for 5 min; the supernatant was subjected to absorbance determination at 412 nm. For the reactive sulfhydryl content determination, the same process was followed that the urea in buffer solution was replaced with PBS (Phosphate-Buffered Saline). The formula to sulfhydryl content was as follows:

(6) $$Sulfhydryl\;content\;(nmol/mgMP)=\frac{A\times D\times 10^{6}}{\varepsilon \times C}$$

where *A* is absorbance value at 412 nm; *ε* is absorbance coefficient (13,600 M^−l^·cm^−l^); *D* is dilution factor; and *C* is protein concentration (mg/mL).

### 2.15. Emulsion Stability Index (ESI) and Emulsifying Activity Index (EAI)

The determination of ESI and EAI was conducted following the method of Zhang et al. [31] with minor modification. Protein solution (8 mL; 1 mg/mL) and soybean oil (2 mL) were mixed using homogenizer (20,000 r/min for 1 min). The bottom emulsion (20 μL) was diluted to 5 mL with SDS (Sodium dodecyl sulfate) (0.1%); after vertexing vigorously for 10 s, the absorbance (*A*_0_) was measured at 500 nm. Again, after 10 min, the bottom emulsion (20 μL) was diluted to 5 mL with SDS (0.1%) and the absorbance (*A*_10_) was determined following the same procedure. The blank control was SDS solution (0.1%). Equations (7) and (8) were used to calculate ESI and EAI, respectively.

(7) $$ESI\;(\%)=\frac{A_{10}}{A_{0}}\times 100$$

(8) $$EAI\;(m^{2}/g)=\frac{2\times 2.303}{C(1-\varphi )\times 10^{4}}\times A_{0}\times di$$

where *C* is the pre-emulsification protein content (g/mL); *φ* is the volume fraction of oil phase in emulsion (*v*/*v*); and *di* is dilution factor.

### 2.16. Statistical Analysis

Triplicate measurements were conducted for all the experiments, and the number of samples per each test group is five. The obtained data were subjected to statistical analysis using SPSS Ver. 26.0 (SPSS Inc., Chicago, IL, USA) and Origin Ver. 9.5 (Origin-Lab Inc., Northampton, MA, USA). The values were presented as average of the three determinations ± SD (standard deviations). One-way ANOVA (analysis of variance) as well as Duncan’s multiple-range test were performed for the determination of significant differences (at 95% confidence level).

## 3. Results and Discussion

### 3.1. Freezing Curves

**Figure 2:**
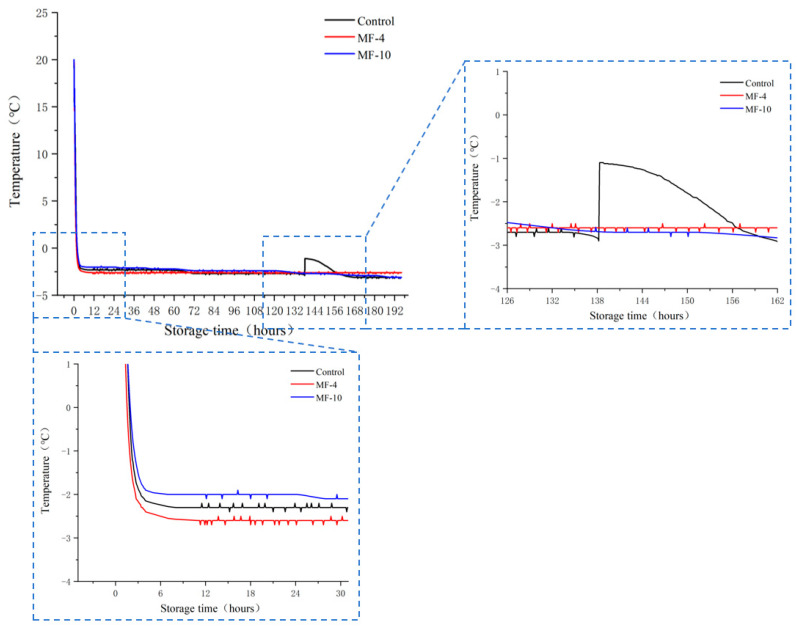
Temperature-time curves of pork tenderloin under different freezing storage conditions. Control: the storage without magnetic field; MF-4: 4 mT static magnetic field-assisted super-chillling storage; MF-10: 10 mT static magnetic field-assisted super-chilling storage.

Temperature profiles of pork samples under various magnetic fields during super-chilling storage are illustrated in Figure 2. The freezing process of pork was evaluated using two parameters: freezing rate and initial nucleation temperature. The freezing rate is the rate at which the sample is reduced from the initial temperature to the initial equilibrium temperature. According to Tang et al. [32], the initial nucleation temperature is the lowest temperature a sample can reach before ice crystals appear. It was demonstrated that the samples subjected to 4 mT magnetic field had a faster freezing rate than the control, with an equilibrium temperature of −2.6 °C, which was remarkably lower than the control value of −2.3 °C, whereas the samples under the magnetic field of 10 mT had a slower freezing rate than the control, and the equilibrium temperature was −2 °C, which was slightly higher than the control value. Meanwhile, the control temperature was reduced to −2.6 °C at 62.4 h during the storage, and a sudden change in temperature was observed at 138 h. Moreover, the initial nucleation temperature was −2.9 °C, after which the sample froze after the formation of ice nuclei with an expense of water.

In addition, the sample temperature under the 4 mT magnetic field decreased to an equilibrium point at the beginning of storage, which did not change markedly throughout the latter storage period. However, the sample temperature slowly decreased throughout the storage period under the 10 mT magnetic field, and it was lower than that with the 4 mT magnetic field freezing at 132.2 h.

Kaale et al. [33] indicated a slow rate of super-chilling resulted in larger ice crystal formation, while a fast super-chilling resulted in smaller ice crystals and a more uniform ice crystal size [19]. Moreover, the presence of magnetic field during super-chilling inhibited the phase transition of water molecules. Lin et al. [34] found that in the presence of a magnetic field, beef was successfully preserved at −4 °C without ice nucleation, reducing the sample freezing temperature.

In our research, the water molecules of the control group are unstable, so phase transitions occurred, and the ice crystals formed were larger. The sudden increase in the temperature of the control group was due to the release of latent heat during the formation of ice [35], and then the temperature gradually fell into equilibrium. However, the ice crystals of the MF-4 group are finer and more uniform, the cooling rate is faster in the initial stage of storage, and the temperature is more stable during storage.

The use of a magnetic field has been found to affect the hydrogen bond network of water molecules [16,17], suggesting that the magnetic field breaks the water hydrogen bonds, which is thought to weaken the hydrogen bonds of large water clusters, splitting into smaller clusters with strong intercluster hydrogen bonds [36], resulting in an increment in the nonfreezable bound water [37]. The use of external magnetic field has been found to reduce Gibbs free-energy, lowering the phase transition driving force [38], which causes the radius of the formed ice nuclei to be smaller and hence less damage to sample tissues.

### 3.2. Drip Loss, WHC, and Cook Loss

**Figure 3:**
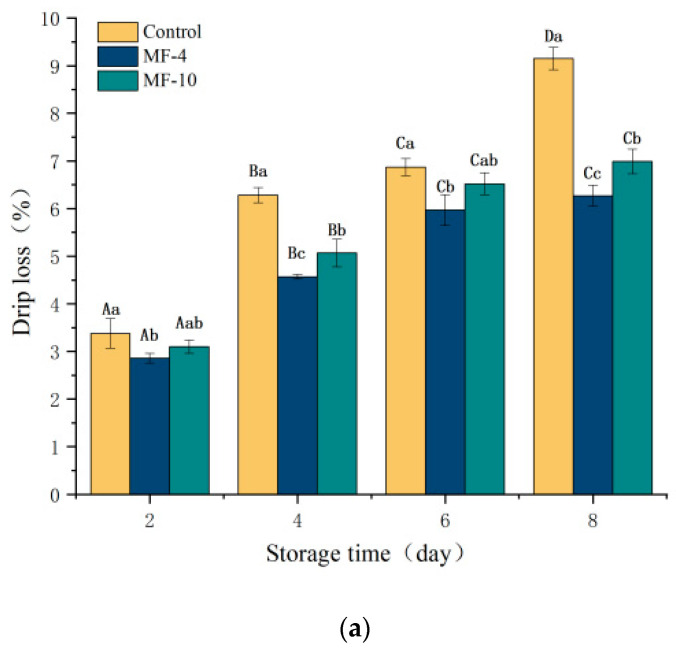

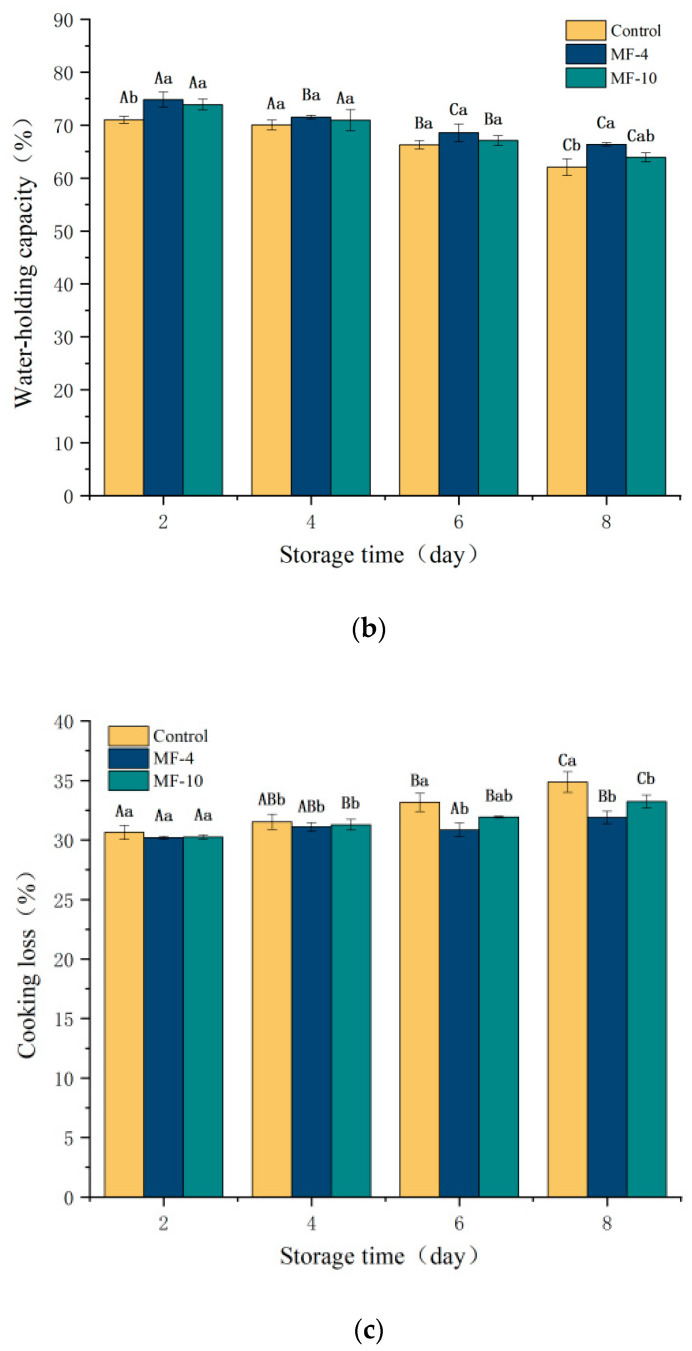
Physicochemical properties of pork tenderloin at 2, 4, 6, and 8 days of storage. (**a**) Drip loss; (**b**) water holding capacity; (**c**) cook loss. Control: the storage without magnetic field; MF-4: 4 mT static magnetic field-assisted freezing storage; MF-10: 10 mT static magnetic field-assisted freezing storage. Different lower case letters (a–c) indicate significant differences (*p* < 0.05) between treatments at the same storage time; and different upper case letters (A–D) indicate significant differences (*p* < 0.05) between different treatment times for the same treatment.

With the prolonged storage duration, drip loss value gradually increased. After 8 days of storage, the drip loss value under the magnetic field was significantly (*p* < 0.05) lower than the control value (Figure 3a). More specifically, the control value was 9.15%, while MF-4 and MF-10 group had, respectively, 2.88% and 2.16%. The moisture of MF-4 and MF-10 remained stable throughout the storage period, while the control underwent phase transition, and then more ice crystals were formed. Ice crystals destroy the fiber interior and microstructure [39], and the free water in the interstices increases after thawing, reducing the amount of water retained by the capillary force, thus leading to an increase in drip loss. Meanwhile, cell membranes are disrupted, leading to an outflow of intracellular water [40], which ultimately results in an increase in drip loss. Lin et al. [34] demonstrated that the freezing of beef tissue at −4 °C with an assistance of static MFs reduced the drip loss.

WHC reflects the capacity to hold the original moisture under external stimuluses; it is one of the important meat quality characteristics. As illustrated in Figure 3b, the WHC decreased with the storage duration; during the storage, the samples maintained a higher WHC under the magnetic field. The values in the MF-4 and MF-10 groups on day eight were 4.29% and 1.85% higher than the controls, respectively. The ice crystals disrupt the meat microstructure, separating the bound water from proteins and leading to protein aggregation and its denaturation, while these ice crystals increase the intracellular solute concentration, again accelerating protein denaturation, both phenomena result to lowering of WHC [41]. The higher WHC of magnetic field groups may be due to the inhibition of ice crystal formation under the action of magnetic field. Similarly, Gan et al. [19] also noted that a specific level magnetic field reduced the ice crystal size, thereby delaying the sample’s WHC decline to some extent.

As illustrated in Figure 3c, the cook loss of the samples treated with magnetic field was lower than the control value. In addition, the magnetic field of 4 mT was more effective than that of 10 mT, respectively, showing 2.96% and 1.63% lower cook loss than that of the control, because of the inhibiting effect on large ice crystals under the magnetic field. The ice crystals have been found to disrupt the muscle tissue structure during freezing, which alters the binding state of proteins to water molecules, leading to a higher cook loss [42]. Similarly, Liu et al. [16] also indicated that the cook loss of pork was markedly reduced under the application of magnetic field during freezing.

### 3.3. MRI Analysis

**Figure 4:**
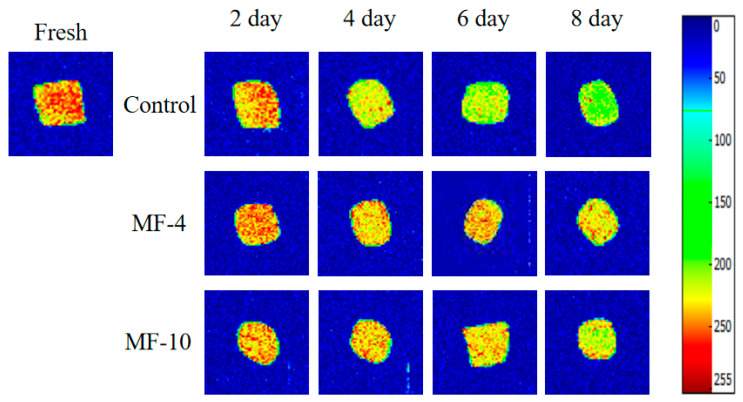
Magnetic resonance images of pork tenderloin at different storage conditions. Control: the storage without magnetic field; MF-4: 4 mT static magnetic field-assisted freezing storage; MF-10: 10 mT static magnetic field-assisted freezing storage.

The assessment of moisture distribution in meat was conducted based on magnetic resonance imaging [43]. Pseudo-color images of pork tenderloin are illustrated in Figure 4. In these images, red mark represents a high density of proton signals and blue represents a low density; meanwhile, a higher density of proton signals indicates a higher water content in the sample [44]. By comparing the signal intensities under different storage conditions, it was found that there was a distinct difference between the control and those stored under the magnetic field. With an increase in storage time, the signal density was observed to decrease in all the sample groups; a gradual decline in red color of the pseudo-color images indicated a gradual decrease in moisture content. However, the magnetic field-assisted super-chilling reduced water loss during storage and the water holding capacity was higher under the 4 mT applied magnetic field, which was similar with the changes in WHC mentioned in the previous section.

### 3.4. Color Measurement

The color of meat is an important quality characteristic, influencing the purchasing potential of consumers. The effect of static MFs at −3 °C on color parameters of pork tenderloin were observed; they included L* value (light-dark), a* value (red-green), b* value (yellow-blue), and Δ*E* value (total color difference) (Table 1). There was an overall decreasing trend in L* value and darkening of meat color with an increasing storage period, while freezing under the magnetic field delayed the decrease in L* value (*p* < 0.05). Additionally, the a* value tended to decrease under different storage conditions. The applied magnetic field delayed the decrease in a* values, when compared with the control; the control, MF-4, and MF-10 groups had an a* value of 5.13, 5.69, and 5.27, respectively. The decrease in a* value might be due to an increase in oxidation during storage and a gradual change in color to dark brown [45]. The Δ*E* value of pork tenderloin subjected to freezing under the magnetic field was significantly lower than the control value, indicating that the color was closer to the fresh sample. In addition, the 4 mT magnetic field had a better color maintenance effect. On the first, fourth, sixth, and eighth day of freezing storage, the Δ*E* value was 18.46%, 39.73%, 55.24%, and 53.33% lower than the control, respectively.

### 3.5. Textural Analysis

Texture properties of pork tenderloin after cooking are illustrated in Table 2; the parameters of hardness, springiness, cohesiveness, resilience, and chewiness were observed. Ice crystals formed during the freezing process of meat cause an increase in the tissue gaps [46], which cause damage to the myofibrillar structure, resulting in an increased hardness after the storage period [47]. The hardening of pork tenderloin increased significantly during the storage period under the magnetic field condition; this indicator was significantly (*p* < 0.05) lower than the control value. It was less severe in the MF-4 group than in the MF-10 group. The resilience and chewiness of all samples increased slightly with the storage duration; however, no significant difference (*p* > 0.05) was observed in the springiness and chewiness of the pork under different magnetic fields. Similarly, no significant difference (*p* > 0.05) was noted for cohesiveness and springiness among the control and magnetic field groups.

### 3.6. pH Analysis

**Figure 5:**
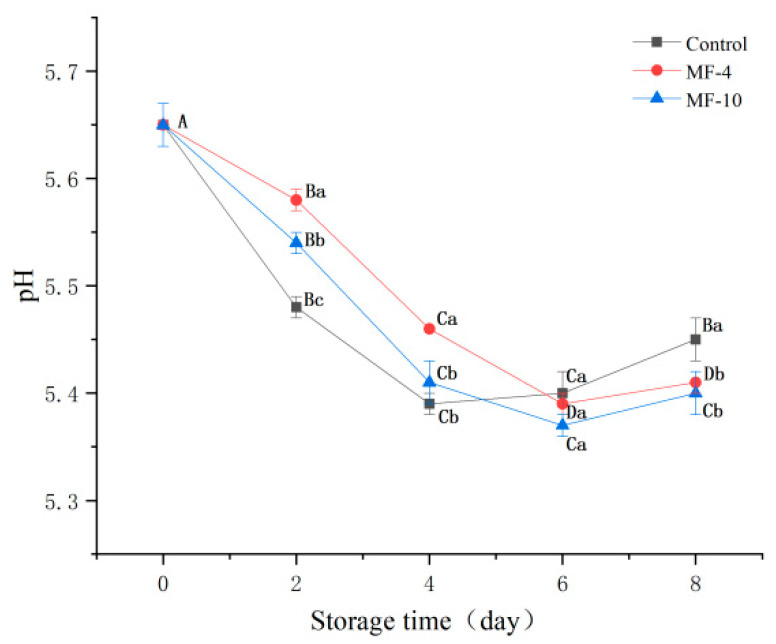
pH analysis of pork tenderloin at 0, 2, 4, 6, and 8 d. Control: no magnetic field storage; MF-4: 4 mT magnetic field-assisted freezing storage; MF-10: 10 mt magnetic field-assisted freezing storage. Different lowercase letters (a–c) indicated significant difference between the same storage time (*p* < 0.05); Different capital letters (A–D) indicated that the difference between the same treatment and different treatment time was statistically significant (*p* < 0.05).

pH value is often used to evaluate the freshness of meat; the pH value of pork during storage is shown in Figure 5. During the super-chilling storage, the pH value of pork in all groups decreased first and then increased. The pH value decreased at the initial stage of storage, which may be due to the anaerobic metabolism of glycogen decomposition to produce lactic acid [48,49], and the decomposition of ATP and creatine phosphate to produce acidic substances [27]. In the process of growth and metabolism, microorganisms can decompose nutrients such as sugars into organic acids [50], which will lead to a decrease in pH value. In addition, the gradual growth of ice crystals during storage will cause damage to muscle tissue, lead to protein denaturation, and then release hydrogen ions [34], which will also reduce pH value. The pH value of each group of pork increased at the late stage of super-chilling storage, which may be due to the decomposition of proteins into ammonia, amines, and alkaline substances under the action of endogenous enzymes and microorganisms in pork [34,51]. On day two, the pH value of MF-4 and MF-10 samples decreased more slowly than that of the control group, and the decline rate increased only on day four. On day eight, the pH value of the control group was 5.45, and the pH value of the MF-4 and MF-10 samples was 5.41 and 5.4, respectively, which was significantly lower than that of the control group (*p* < 0.05). This may be because there are fewer microorganisms in the MF-4 and MF-10 samples, which are less able to break down nutrients.

### 3.7. TVBN Analysis

**Figure 6:**
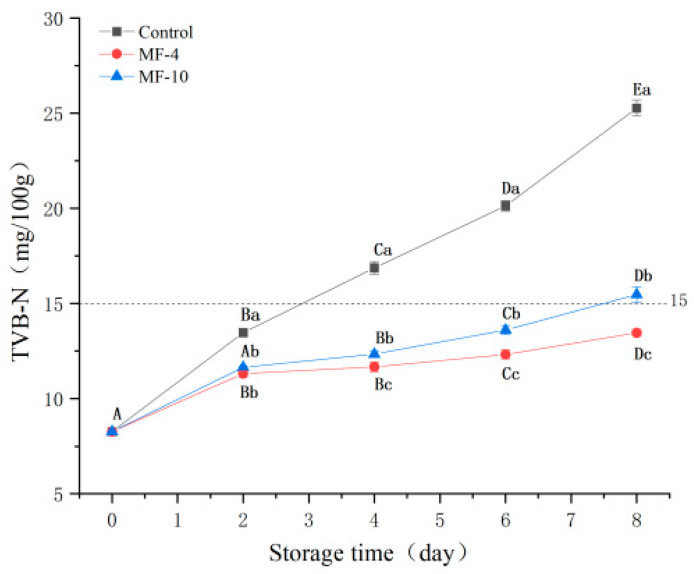
TVB-N of pork tenderloin at 0, 2, 4, 6, and 8 days of storage. Control: the storage without magnetic field; MF-4: 4 mT static magnetic field-assisted freezing storage; MF-10: 10 mT static magnetic field-assisted freezing storage. Different lower case letters (a–c) indicate significant differences (*p* < 0.05) between treatments at the same storage time; different upper case letters (A–E) indicate significant differences (*p* < 0.05) between different treatment times for the same treatment.

TVBN is an indicator for freshness evaluation of meat [52]; its values in tested samples are shown in Figure 6a. The TVBN values of all the samples showed an increasing trend throughout this period, which was attributed to the breakdown of proteins by spoilage microbiota during super-chilled storage [53]. The TVBN value of fresh pork sample was 8.27 mg/100 g; after 8 days of super-chilled storage, its value was 25.27, 13.46, and 15.47 mg/100 g, respectively, for the control sample, MF-4 group, and MF-10 group. The TVBN value of the control was reported to exceed the threshold value of 15 mg/100 g [34] on day four of storage (16.87 mg/100 g), whereas the super-chilled storage under the magnetic field condition significantly prolonged the freshness period of the pork. Simultaneously, Zhang et al. [27] found that the freezing under alternating magnetic field at 2 mT was able to retard the TVBN increment in beef.

### 3.8. Solubility of MPs

**Figure 7:**
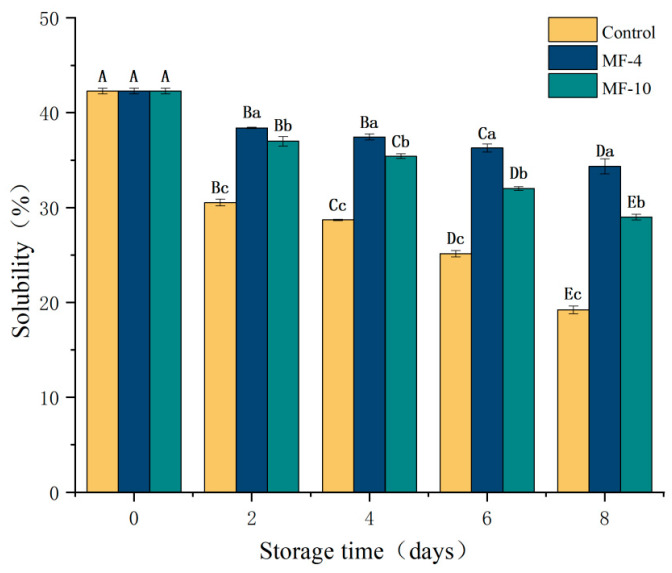
Solubility of pork tenderloin at different storage conditions and durations. Control: the storage without magnetic field; MF-4: 4 mT static magnetic field-assisted freezing storage; MF-10: 10 mT static magnetic field-assisted freezing storage. Different lower case letters (a–c) indicate significant differences (*p* < 0.05) between treatments at the same storage time; and different upper case letters (A–E) indicate significant differences (*p* < 0.05) between different treatment times for the same treatment.

Protein solubility refers to its hydration property and one of the indicators of protein denaturation, which affects the protein functionalities. The solubility of MPs in the control and MF-4 groups showed an overall decreasing trend with increasing storage duration (Figure 7). On 2, 4, 6 and 8 days of storage, MP solubility decreased in sequence to 42.28% to 38.4%, 37.44%, 36.29%, and 34.35%, respectively, in the MF-4 group, while MP solubility decreased to 37%, 35.42%, 32.02%, and 29.01%, respectively, in the MF-10 group, indicating significantly higher values than the control (28.71%, 28.71%, 25.14% and 19.23%, respectively). These results suggested that the magnetic field was able to reduce the degree of denaturation of proteins in pork. The decrease in solubility during storage might be due to the ice crystal formation, which disrupted the structure of myofibrillar proteins, leading to their denaturation [54]. In addition, it may also be because the cooling rate of the control group is slow, and interaction forces among molecules (for example, hydrogen bond, disulfide bond, and hydrophobic bond) caused conformational changes in proteins, which on the one hand increased the degree of aggregation of proteins once the hydrophobic groups were exposed, while on another hand, the increased hydrophobicity of proteins resulted in a decrease in solubility [54,55].

### 3.9. Reactive and Total Sulfhydryl Content of MPs

**Figure 8:**
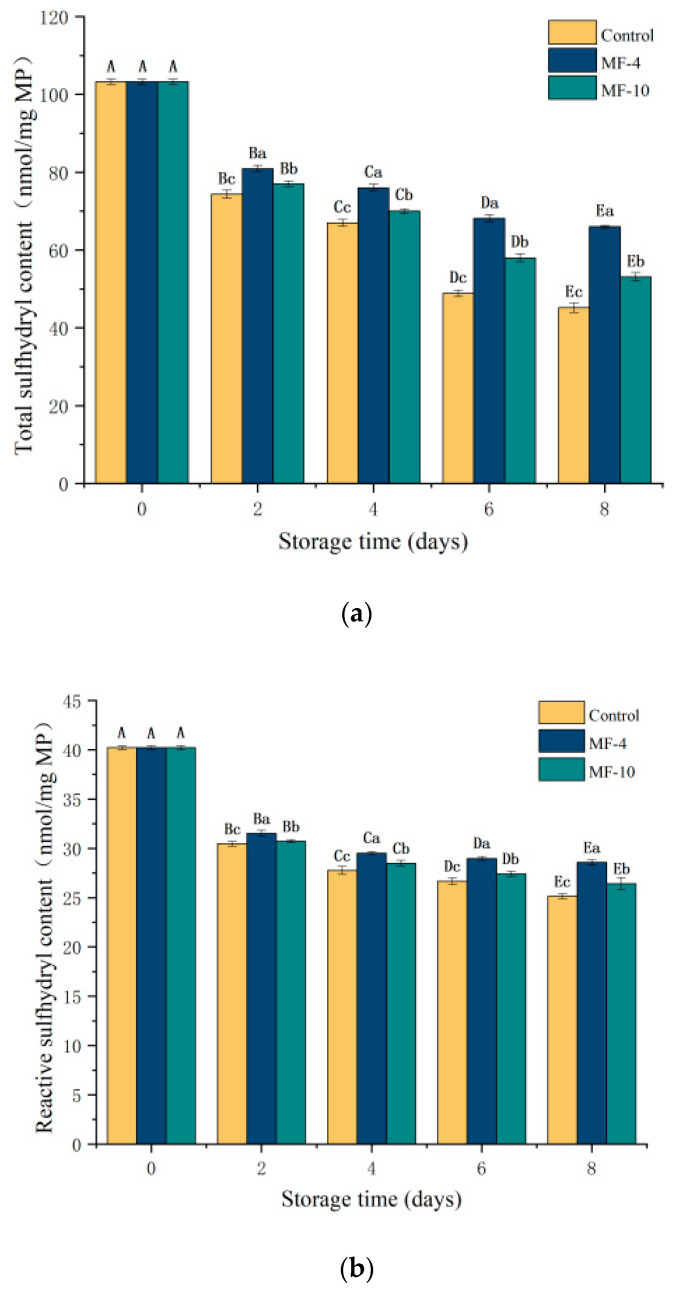
Total and reactive sulfhydryl content of MPs in pork tenderloin at different storage conditions and durations. (**a**) Total sulfhydryl content; (**b**) active sulfhydryl content. Control: super-chilled storage without magnetic field; MF-4: 4 mT static magnetic field-assisted freezing storage; MF-10: 10 mT static magnetic field-assisted freezing storage. Different lower case letters (a–c) indicate significant differences (*p* < 0.05) between treatments at the same storage time; and different upper case letters (A–E) indicate significant differences (*p* < 0.05) between different treatment times for the same treatment.

Changes in the content of sulfhydryl groups are indicators of protein folding and disulfide bond formation, as well as protein conformation changes that reflect the extent of protein oxidation [56]. As illustrated in Figure 8a, the total sulfhydryl content was decreasing with storage time. The content of fresh samples was 103.26 nmol/mg MP, and on the eighth day of storage, the content of the control group was 45.16 nmol/mg MP, compared to 65.99 nmol/mg MP and 53.11 nmol/mg MP for the MF-4 and MF-10 groups, respectively. Similar findings of a decrease in total sulfhydryl content were observed by Jiang et al. [57] during the freezing of grass carp. The reactive sulfhydryl groups are located on the protein surface and are exposed, which are important for maintaining the tertiary and quaternary structure of proteins [58]. Figure 8b shows the active sulfhydryl content during the storage. It was significantly reduced on the second day, with a smaller reduction thereafter and consistently higher in MF-4 than the control value. The active sulfhydryl content was 25.14 nmol/mg MP, 28.58 nmol/mg MP, and 26.42 nmol/mg MP for the control sample, MF-4 group, and MF-10 group, respectively, on the eighth day. During freezing, reactive sulfhydryl groups are oxidized and disulfide bonds are formed, decreasing the total sulfhydryl content; and protein aggregation causes sulfhydryl groups to be hidden, so there was a reduction in total sulfhydryl content [59,60]. The lower sulfhydryl value in the control might be due to the formation of larger ice crystals, causing a greater alteration in the spatial structure of the MP; then, disulfide bonds were formed after the exposure and oxidation of sulfhydryl groups [61].

### 3.10. Emulsion Stability (ESI) and Emulsifying Activity (EAI) of MPs

**Figure 9:**
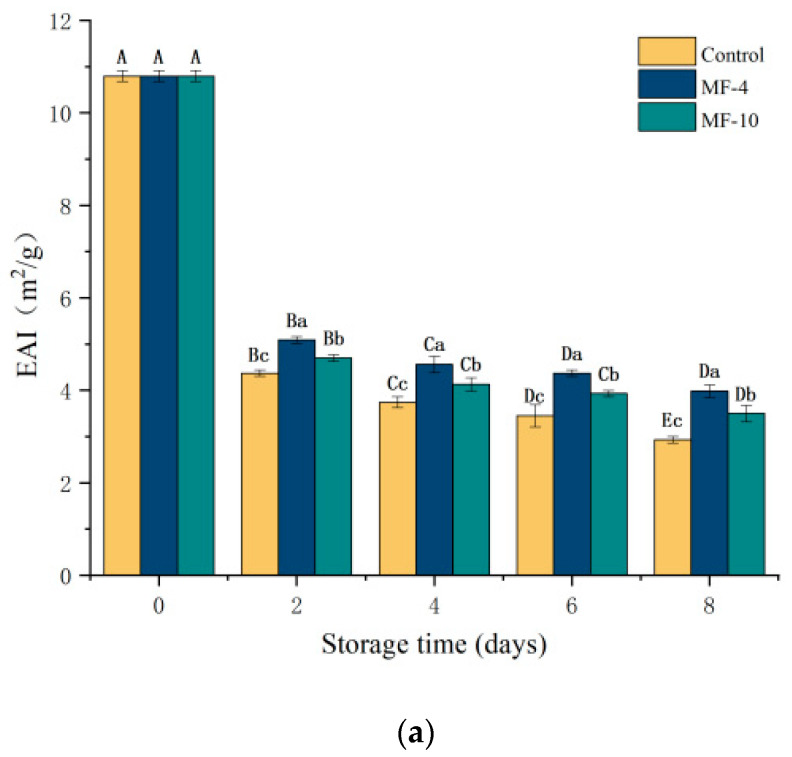

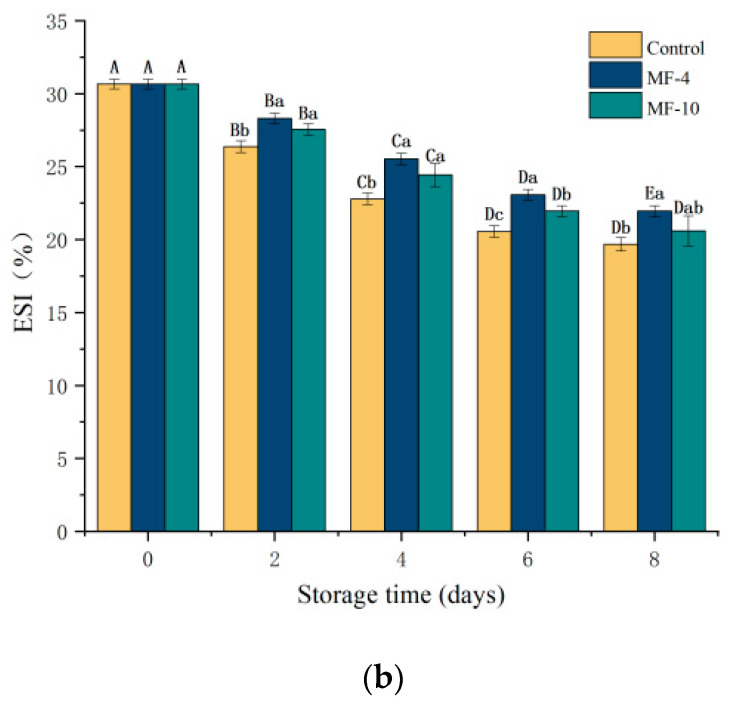
Emulsifying activity (EAI) and emulsion stability (ESI) of MPs in pork tenderloin at different storage conditions and durations. (**a**) Emulsifying activity index; (**b**) emulsion stability index. Control: the storage without magnetic field; MF-4: 4 mT static magnetic field-assisted freezing storage; MF-10: 10 mT static magnetic field-assisted freezing storage. Different lower case letters (a–c) indicate significant differences (*p* < 0.05) between treatments at the same storage time; and different upper case letters (A–E) indicate significant differences (*p* < 0.05) between different treatment times for the same treatment.

The ESI and EAI reflect the stability of protein emulsions [62]. The EAI refers to the interfacial area, stabilized by a protein unit weight, which reflects the protein adsorption capacity at the water–oil interface [63]. Figure 9a,b, respectively, present the changes in EAI and ESI of the pork under different storage conditions. Both the EAI and ESI indicated a decreasing trend with storage period, and those of the controls were lower than the MF groups. The EAI was 2.93 m^2^/g, 3.98 m^2^/g, and 3.5 m^2^/g, while the ESI was 19.68%, 21.96%, and 20.6% for the control sample, MF-4 group, and MF-10 group, respectively, on the eighth day of storage. In the case of the control samples, the myofibrillar proteins were damaged and were denatured to a large extent owing to the ice crystal formation. The disulfide bonds formed along with the oxidation reactions destabilized the protein conformation, making it difficult to form stable emulsions [64]. The denaturation of proteins and formation of larger protein aggregates resulted in fewer proteins adsorbed at the water–oil interface, decreasing the protein flexibility, which were adsorbed on the droplet surface of the oil, which led to a lower EAI and ESI value of myofibrillar proteins [31,65]. Guo et al. [62] indicated that higher myosin solubility allows faster protein diffusion into the water–oil interface, which favors EAI. This study illustrated that the MF group had a higher solubility than the control group, which corresponded to a higher EAI level of the MF group, indicating that the magnetic field inhibited the oxidation and denaturation of proteins to a certain degree.

## 4. Conclusions

This work investigated that SMF could affect the freezing characteristics, physicochemical properties, and myofibrillar protein characteristics of pork during storage. The applying static MFs of 4 mT and 10 mT were conducive to the meat quality under super-chilled storage. The initial equilibrium temperature of the pork at 4 mT static MF was −2.3 °C, which was 0.3 °C less than the control value. There was no freezing phenomenon on the pork at −3 °C storage temperature when subjected to the magnetic field, whereas in the control group, the phase transition was obvious and the samples were frozen. The magnetic field was found to have a positive effect on WHC and cook loss under super-chilled storage; in addition, it significantly reduced drip loss. The MRI results indicated a significant reduction in water loss. SMF assisted the pork tenderloin in retaining its color, and inhibited the increase in TVB-N content. In terms of texture, SMF delayed the hardening of cooked samples during storage, and the impact was more pronounced in the MF-4 group. The use of a magnetic field also delayed the reduction in myofibrillar protein solubility, emulsifying activity, emulsion stability, and sulfhydryl content. In conclusion, SMF treatment is a promising and effective assistive method to improve the super-chilling quality of pork.

## Figures and Tables

**Table 1:** L*, a*, b*, and Δ*E* values of pork tenderloin at different storage conditions and durations. Control: the storage without magnetic field; MF-4: 4 mT static magnetic field-assisted freezing storage; MF-10: 10 mT static magnetic field-assisted freezing storage.

Samples	Color Parameters	Fresh	2 Day	4 Day	6 Day	8 Day
Control	L*	50.0 ± 0.2A	47.2 ± 0.4Bb	47 ± 0.9BCb	46.3 ± 1.4BCb	45.2 ± 0.4Db
	a*	6.7 ± 0.2A	6.0 ± 0.2Ba	5.6 ± 0.2BCb	5.5 ± 0.5BCa	5.1 ± 0.2Db
	b*	6.1 ± 0.1A	4.7 ± 0.0BCa	4.7 ± 0.0BCa	4.6 ± 0.1Ca	4.8 ± 0.0Bab
	Δ*E*		3.3 ± 0.3Aa	3.6 ± 0.8ABa	4.3 ± 1.1ABa	5.1 ± 0.3Ba
MF-4	L*	50.0 ± 0.2A	48.7 ± 0.4BCa	49.0 ± 0.6Ba	48.5 ± 0.1BCa	47.8 ± 0.1Ca
	a*	6.7 ± 0.2A	6.3 ± 0.1ABa	6.6 ± 0.4Aa	5.8 ± 0.4Ba	5.7 ± 0.1Ba
	b*	6.1 ± 0.1A	4.5 ± 0.1Ca	4.7 ± 0.2BCa	4.6 ± 0.1Ca	5.0 ± 0.3Ba
	Δ*E*		2.7 ± 0.2ABb	2.1 ± 0.3Ab	1.9 ± 0.5ABb	2.4 ± 0.1Bb
MF-10	L*	50.0 ± 0.2A	48.4 ± 0.7Ba	48.5 ± 0.4Bab	48.0 ± 0.5Bab	47.7 ± 0.2Bab
	a*	6.7 ± 0.2A	5.9 ± 0.2ABa	5.2 ± 0.3Bb	5.6 ± 0.8Ba	5.3 ± 0.1Bb
	b*	6.1 ± 0.1A	4.8 ± 0.3Ba	4.6 ± 0.1Ba	4.5 ± 0.1Ba	4.5 ± 0.1Bb
	Δ*E*		2.3 ± 0.4Ab	2.6 ± 0.4ABab	2.9 ± 0.3ABab	3.1 ± 0.1Bb

Different lower case letters (a,b) indicate significant differences (*p* < 0.05) between treatments at the same storage time; and different upper case letters (A–D) indicate significant differences (*p* < 0.05) between different treatment times for the same treatment.

**Table 2:** Textural parameters of pork tenderloin at different storage conditions and durations. Control: the storage without magnetic field; MF-4: 4 mT static magnetic field-assisted freezing storage; MF-10: 10 mT static magnetic field-assisted freezing storage.

Samples	Texture Parameters	Fresh	2 Day	4 Day	6 Day	8 Day
Control	Hardness (N)	54.1 ± 3.1A	60.9 ± 1.4Ba	68.3 ± 1.4Ca	72.6 ± 1.4Da	76.3 ± 1.3Da
	Springiness	0.7 ± 0.0A	0.7 ± 0.0ABa	0.8 ± 0.0ABb	0.8 ± 0.0Ba	0.8 ± 0.0Ba
	Cohesiveness	0.7 ± 0.0A	0.7 ± 0.0Aa	0.7 ± 0.0Aa	0.7 ± 0.0Ba	0.7 ± 0.0Ba
	Resilience	0.3 ± 0.0A	0.3 ± 0.0Aa	0.3 ± 0.0ABa	0.4 ± 0.0Ba	0.4 ± 0.0Ba
	Chewiness	2686.5 ± 159.2A	2866.0 ± 149.3Ba	3554.3 ± 113.8Ca	4277.9 ± 303.2Da	4071.8 ± 305.3Da
MF-4	Hardness (N)	54.1 ± 3.1A	61.8 ± 1.5Ba	64.9 ± 1.0BCa	66.5 ± 2.9BCb	69.6 ± 1.7Cb
	Springiness	0.7 ± 0.0A	0.8 ± 0.0Ba	0.8 ± 0.0ABb	0.8 ± 0.0ABa	0.8 ± 0.0ABb
	Cohesiveness	0.7 ± 0.0A	0.7 ± 0.0Aa	0.6 ± 0.0Ab	0.7 ± 0.0Ba	0.7 ± 0.0Ab
	Resilience	0.3 ± 0.0A	0.3 ± 0.0Aa	0.3 ± 0.0Ab	0.3 ± 0.0Ba	0.3 ± 0.0Ba
	Chewiness	2686.0 ± 159.2A	3219.0 ± 65.0Ba	3690.9 ± 49.4Bb	3446.2 ± 480.6BCb	3691.8 ± 40.3Cb
MF-10	Hardness (N)	54.1 ± 3.1A	63.7 ± 1.1Ba	65.4 ± 1.9BCa	68.6 ± 1.4CDab	72.5 ± 1.5Dab
	Springiness	0.7 ± 0.0A	0.8 ± 0.0ABa	0.8 ± 0.0Ba	0.8 ± 0.0ABa	0.8 ± 0.0Ba
	Cohesiveness	0.7 ± 0.0A	0.7 ± 0.0Aa	0.7 ± 0.0Aab	0.7 ± 0.0Ba	0.7 ± 0.0Ba
	Resilience	0.3 ± 0.0A	0.3 ± 0.0Aa	0.3 ± 0.0Aa	0.3 ± 0.0Aa	0.4 ± 0.0Ba
	Chewiness	2686.5 ± 159.2A	3186.5 ± 170.7Ba	3501.7 ± 27.5BCa	3599.9 ± 130.7Cab	4002.1 ± 277.2Da

Different lower case letters (a,b) indicate significant differences (*p* < 0.05) between treatments at the same storage time; and different upper case letters (A–D) indicate significant differences (*p* < 0.05) between different treatment times for the same treatment.

## Data Availability

The original contributions presented in the study are included in the article, further inquiries can be directed to the corresponding author.

## References

[1] Duun A.S., Rustad T. (2007). Quality changes during superchilled storage of cod (*Gadus morhua*) fillets. Food Chem..

[2] Kaale L.D., Eikevik T.M. (2013). A histological study of the microstructure sizes of the red and white muscles of Atlantic salmon (Salmo solar) fillets during superchilling process and storage. J. Food Eng..

[3] Magnussen O.M., Haugland A., Hemmingsen A.K.T., Johansen S., Nordtvedt T.S. (2008). Advances in superchilling of food—Process characteristics and product quality. Trends Food Sci. Technol..

[4] Kaale L.D., Eikevik T.M. (2014). The development of ice crystals in food products during the superchilling process and following storage, a review. Trends Food Sci. Technol..

[5] Gallart-Jornet L., Rustad T., Barat J.M., Fito P., Escriche I. (2007). Effect of superchilled storage on the freshness and salting behaviour of Atlantic salmon (*Salmo salar*) fillets. Food Chem..

[6] Kim H., Hong G.-P. (2022). Comparison of superchilling and supercooling on extending the fresh quality of beef loin. Foods.

[7] Liang C., Zhang D., Zheng X., Wen X., Yan T., Zhang Z., Hou C. (2021). Effects of different storage temperatures on the physicochemical properties and bacterial community structure of fresh lamb meat. Food Sci. Anim. Resour..

[8] Park D.H., Lee S., Lee J., Kim E.J., Jo Y.-J., Kim H., Choi M.-J., Hong G.-P. (2021). Stepwise cooling mediated feasible supercooling preservation to extend freshness of mackerel fillets. LWT-Food Sci. Technol..

[9] Park D.H., Lee S., Kim E.J., Jo Y.-J., Choi M.-J. (2022). Development of a stepwise algorithm for supercooling storage of pork belly and chicken breast and its effect on freshness. Foods.

[10] Pomponio L., Ruiz-Carrascal J. (2017). Oxidative deterioration of pork during superchilling storage. J. Sci. Food Agric..

[11] Lu X., Zhang Y., Zhu L., Luo X., Hopkins D.L. (2019). Effect of superchilled storage on shelf life and quality characteristics of *M. longissimus lumborum* from Chinese Yellow cattle. Meat Sci..

[12] Pomponio L., Bukh C., Ruiz-Carrascal J. (2018). Proteolysis in pork loins during superchilling and regular chilling storage. Meat Sci..

[13] Lu X., Zhang Y., Xu B., Zhu L., Luo X. (2020). Protein degradation and structure changes of beef muscle during superchilled storage. Meat Sci..

[14] Bahuaud D., Morkore T., Langsrud O., Sinnes K., Veiseth E., Ofstad R., Thomassen M.S. (2008). Effects of −1.5 °C Super-chilling on quality of Atlantic salmon (*Salmo salar*) pre-rigor Fillets: Cathepsin activity, muscle histology, texture and liquid leakage. Food Chem..

[15] Leygonie C., Britz T.J., Hoffman L.C. (2012). Impact of freezing and thawing on the quality of meat: Review. Meat Sci..

[16] Liu F., Yang N., Zhang L., Jin Y., Jin Z., Xu X. (2023). Effect of weak magnetic field on the water-holding properties, texture, and volatile compounds of pork and beef during frozen storage. Food Biosci..

[17] Lu N., Ma J., Sun D.W. (2022). Enhancing physical and chemical quality attributes of frozen meat and meat products: Mechanisms, techniques and applications. Trends Food Sci. Technol..

[18] Tang J., Zhang H., Tian C., Shao S. (2020). Effects of different magnetic fields on the freezing parameters of cherry. J. Food Eng..

[19] Gan S., Zhang M., Jiang Q. (2024). Pork freezing and quality improvement: The effect of immersion freezing assisted by magnetic field. Food Bioprocess Technol..

[20] Zhang L., Yang Z., Deng Q. (2021). Effects of pulsed magnetic field on freezing kinetics and physical properties of water and cucumber tissue fluid. J. Food Eng..

[21] You Y., Her J.-Y., Shafel T., Kang T., Jun S. (2020). Supercooling preservation on quality of beef steak. J. Food Eng..

[22] Wang T., Jin Y., Yang N., Xu D., Yang Z., Tan Y., Xu X., Jin Z., Cui B. (2022). Effect of magnetic field with different dimensions on quality of avocado puree during frozen storage. Int. J. Food Sci. Technol..

[23] Park D.H., Kim E.J., Kim H., Hong G.-P., Choi M.-J. (2022). Conditions of the stepwise cooling algorithm for stable supercooling preservation and freshness of pork loin. Foods.

[24] Jia F., Jing Y., Dai R., Li X., Xu B. (2020). High-pressure thawing of pork: Water holding capacity, protein denaturation and ultrastructure. Food Biosci..

[25] Cheng S., Wang X., Li R., Yang H., Wang H., Wang H., Tan M. (2019). Influence of multiple freeze-thaw cycles on quality characteristics of beef semimembranous muscle: With emphasis on water status and distribution by LF-NMR and MRI. Meat Sci..

[26] Pirayesh H., Park B.-D., Khanjanzadeh H., Park H.-J., Cho Y.-J. (2023). Nanocellulose-based ammonia sensitive smart colorimetric hydrogels integrated with anthocyanins to monitor pork freshness. Food Control.

[27] Zhang W., Wang Y., Zhu X., Cui B., Yang N. (2023). Influence of alternating magnetic field on the quality of beef and its protein during cold storage. Int. J. Food Sci. Technol..

[28] Park D., Xiong Y.L., Alderton A.L. (2007). Concentration effects of hydroxyl radical oxidizing systems on biochemical properties of porcine muscle myofibrillar protein. Food Chem..

[29] Du X., Zhao M., Pan N., Wang S., Xia X., Zhang D. (2021). Tracking aggregation behaviour and gel properties induced by structural alterations in myofibrillar protein in mirror carp (*Cyprinus carpio*) under the synergistic effects of pH and heating. Food Chem..

[30] Hou Q., Cheng Y.-p., Kang D.-c., Zhang W.-g., Zhou G.-h. (2020). Quality changes of pork during frozen storage: Comparison of immersion solution freezing and air blast freezing. Int. J. Food Sci. Technol..

[31] Zhang C., Liu H., Xia X., Sun F., Kong B. (2021). Effect of ultrasound-assisted immersion thawing on emulsifying and gelling properties of chicken myofibrillar protein. LWT-Food Sci. Technol..

[32] Tang J., Shao S., Tian C. (2019). Effects of the magnetic field on the freezing parameters of the pork. Int. J. Refrig..

[33] Kaale L.D., Eikevik T.M., Bardal T., Kjorsvik E., Nordtvedt T.S. (2013). The effect of cooling rates on the ice crystal growth in airpacked salmon fillets during superchilling and superchilled storage. Int. J. Refrig..

[34] Lin H., Zhao S., Han X., Guan W., Liu B., Chen A., Sun Y., Wang J. (2022). Effect of static magnetic field extended supercooling preservation on beef quality. Food Chem..

[35] Kiani H., Sun D.-W. (2011). Water crystallization and its importance to freezing of foods: A review. Trends Food Sci. Technol..

[36] Toledo E.J., Ramalho T.C., Magriotis Z.M. (2008). Influence of magnetic field on physical–chemical properties of the liquid water: Insights from experimental and theoretical models. J. Mol. Struct..

[37] Otero L., Rodríguez A.C., P’erez-Mateos M., Sanz P.D. (2016). Effects of magnetic fields on freezing: Application to biological products. Compr. Rev. Food Sci. Food Saf..

[38] Qin Y., Dong B., Li W. (2020). Experimental study of the frosting characteristic of water on a cold surface in the magnetic field. Exp. Therm. Fluid Sci..

[39] Wang Z., Tan Y., Na Y., Jin Y., Sun H., Xu X. (2019). Influence of oscillating uniform magnetic field and iron supplementation on quality of freeze-thawed surimi. RSC Adv..

[40] Leygonie C., Britz T.J., Hoffman L.C. (2012). Meat quality comparison between fresh and frozen/thawed ostrich M. iliofibularis. Meat Sci..

[41] Sun Q., Zhang H., Yang X., Hou Q., Zhang Y., Su J., Liu X., Wei Q., Dong X., Ji H. (2023). Insight into muscle quality of white shrimp (Litopenaeus vannamei) frozen with static magnetic-assisted freezing at different intensities. Food Chem.-X.

[42] Zhou J., Dong X., Kong B., Sun Q., Ji H., Liu S. (2023). Effects of magnetic field-assisted immersion freezing at different magnetic field intensities on the muscle quality of golden pompano (*Trachinotus ovatus*). Food Chem..

[43] Avila M., Caballero D., Antequera T., Luisa Duran M., Caro A., Perez-Palacios T. (2018). Applying 3D texture algorithms on MRI to evaluate quality traits of loin. J. Food Eng..

[44] Cheng S., Li R., Yang H., Wang S., Tan M. (2020). Water status and distribution in shiitake mushroom and the effects of drying on water dynamics assessed by LF-NMR and MRI. Dry. Technol..

[45] Zhao S., Lin H., Li S., Liu C., Meng J., Guan W., Liu B. (2022). Modeling of chilled/supercooled pork storage quality based on the entropy weight method. Animals.

[46] Mousakhani-Ganjeh A., Hamdami N., Soltanizadeh N. (2015). Impact of high voltage electric field thawing on the quality of frozen tuna fish (*Thunnus albacares*). J. Food Eng..

[47] Lopez-Lopez I., Cofrades S., Yakan A., Solas M.T., Jimenez-Colmenero F. (2010). Frozen storage characteristics of low-salt and low-fat beef patties as affected by Wakame addition and replacing pork backfat with olive oil-in-water emulsion. Food Res. Int..

[48] Kaale L.D., Eikevik T.M., Rustad T., Kolsaker K. (2011). Superchilling of food: A review. J. Food Eng..

[49] Liu H., Saito Y., Al Riza D.F., Kondo N., Yang X., Han D. (2019). Rapid evaluation of quality deterioration and freshness of beef during low temperature storage using three-dimensional fluorescence spectroscopy. Food Chem..

[50] Hansen A.A., Moen B., Rodbotten M., Berget I., Pettersen M.K. (2016). Effect of vacuum or modified atmosphere packaging (MAP) in combination with a CO2 emitter on quality parameters of cod loins (*Gadus morhua*). Food Packag. Shelf Life.

[51] Sujiwo J., Kim H.J., Song S.O., Jang A. (2019). Relationship between quality and freshness traits and torrymeter value of beef loin during cold storage. Meat Sci..

[52] Bekhit A., Holman B., Giteru S., Hopkins D. (2021). Total volatile basic nitrogen (TVB-N) and its role in meat spoilage: A review. Trends Food Sci. Technol..

[53] Chang W., Liu F., Sharif H.R., Huang Z., Goff H.D., Zhong F. (2019). Preparation of chitosan films by neutralization for improving their preservation effects on chilled meat. Food Hydrocoll..

[54] Li F., Du X., Wang B., Pan N., Xia X., Bao Y. (2021). Inhibiting effect of ice structuring protein on the decreased gelling properties of protein from quick-frozen pork patty subjected to frozen storage. Food Chem..

[55] Li J., Ma X., Wang Y., Du M., Wang Y., Du J., Li K., Bai Y. (2022). Effects of immersion freezing on the conformational changes of myofibrillar proteins in pork under ultrasonic power densities of 0, 15, 30 and 45 W L^−1^. Int. J. Food Sci. Technol..

[56] Higuera-Barraza O.A., Torres-Arreola W., Ezquerra-Brauer J.M., Cinco-Moroyoqui F.J., Rodriguez Figueroa J.C., Marquez-Rios E. (2017). Effect of pulsed ultrasound on the physicochemical characteristics and emulsifying properties of squid (*Dosidicus gigas*) mantle proteins. Ultrason. Sonochem..

[57] Jiang L., Wu S. (2018). Pullulan suppresses the denaturation of myofibrillar protein of grass carp (*Ctenopharyngodon idella*) during frozen storage. Int. J. Biol. Macromol..

[58] Li L.Y., Bai Y., Cai R.Y., Wu C.L., Wang P., Xu X.L., Sun J. (2018). Alkaline pH-dependent thermal aggregation of chicken breast myosin:Formation of soluble aggregates. CyTA-J. Food.

[59] Gao W., Huang Y., Zeng X.-a., Brennan M.A. (2019). Effect of soluble soybean polysaccharides on freeze-denaturation and structure of myofibrillar protein of bighead carp surimi with liquid nitrogen freezing. Int. J. Biol. Macromol..

[60] Sun Q., Chen Q., Xia X., Kong B., Diao X. (2019). Effects of ultrasound-assisted freezing at different power levels on the structure and thermal stability of common carp (*Cyprinus carpio*) proteins. Ultrason. Sonochem..

[61] Sompongse W., Itoh Y., Obatake A. (1996). Effect of cryoprotectants and a reducing reagent on the stability of actomyosin during ice storage. Fish. Sci..

[62] Guo X., Gao F., Zhang Y., Peng Z., Jamali M.A. (2021). Effect of L-histidine and L-lysine on the properties of oil-in-water emulsions stabilized by porcine myofibrillar proteins at low/high ionic strength. LWT-Food Sci. Technol..

[63] Pearce K.N., Kinsella J.E. (1978). Emulsifying properties of proteins: Evaluation of a turbidimetric technique. J. Agric. Food Chem..

[64] Pan N., Wan W., Du X., Kong B., Liu Q., Lv H., Xia X., Li F. (2022). Mechanisms of change in emulsifying capacity induced by protein denaturation and aggregation in quick-frozen pork patties with different fat levels and freeze–thaw cycles. Foods.

[65] Ma X., Yan T., Hou F., Chen W., Miao S., Liu D. (2019). Formation of soy protein isolate (SPI)-citrus pectin (CP) electrostatic complexes under a high-intensity ultrasonic field: Linking the enhanced emulsifying properties to physicochemical and structural properties. Ultrason. Sonochem..

